# Mathematical modeling for operative improvement of the decoloration of Acid Red 27 by a novel microbial consortium of *Trametes versicolor* and *Pseudomonas putida*: A multivariate sensitivity analysis

**DOI:** 10.1016/j.heliyon.2023.e21793

**Published:** 2023-10-31

**Authors:** L.A. Martínez-Castillo, C.A. González-Ramírez, A. Cortazar-Martínez, J.R. González-Reyes, E.M. Otazo-Sánchez, J.R. Villagómez-Ibarra, R. Velázquez-Jiménez, G.M. Vázquez-Cuevas, A. Madariaga-Navarrete, O.A. Acevedo-Sandoval, C. Romo-Gómez

**Affiliations:** a Universidad Autónoma del Estado de Hidalgo, Área Académica de Química, Instituto de Ciencias Básicas e Ingeniería, Carr. Pachuca-Tulancingo km. 4.5, Col. Carboneras, Mineral de la Reforma, Hidalgo, C.P. 42184, Mexico; b Universidad Autónoma del Estado de Hidalgo, Escuela Superior de Apan, Carr. Apan-Calpulalpan, S/N, Col. Chimalpa Tlalayote, Apan, Hidalgo, C.P. 43920, Mexico; c Investigación Aplicada al Bienestar Social y Ambiental (INABISA), A.C., Río Papagayo S/N, Col. Amp. El Palmar, Pachuca, Hidalgo, C.P. 42088, Mexico; d Universidad Autónoma del Estado de Hidalgo, Área Académica de Ciencias Agrícolas y Forestales, Instituto de Ciencias Agropecuarias, Carr. Tulancingo-Santiago Tulantepec S/N, Tulancingo, Hidalgo, C.P. 43600, Mexico

**Keywords:** Textile wastewater treatment, Mathematical model, Azo dye, Response surface method, Microbial consortium

## Abstract

In this work, it is presented a first approach of a mathematical and kinetic analysis for improving the decoloration and further degradation process of an azo dye named acid red 27 (AR27), by means of a novel microbial consortium formed by the fungus *Trametes versicolor* and the bacterium *Pseudomonas putida*. A multivariate analysis was carried out by simulating scenarios with different operating conditions and developing a specific mathematical model based on kinetic equations describing all stages of the biological process, from microbial growth and substrate consuming to decoloration and degradation of intermediate compounds. Additionally, a sensitivity analysis was performed by using a factorial design and the Response Surface Method (RSM), for determining individual and interactive effects of variables like, initial glucose concentration, initial dye concentration and the moment in time for bacterial inoculation, on response variables assessed in terms of the minimum time for: full decoloration of AR27 (R_1_ = 2.375 days); maximum production of aromatic metabolites (R_2_ = 1.575 days); and full depletion of aromatic metabolites (R_3_ = 12.9 days). Using RSM the following conditions improved the biological process, being: an initial glucose concentration of 20 g l^-1^, an initial AR27 concentration of 0.2 g l^-1^ and an inoculation moment in time of *P. putida* at day 1. The mathematical model is a feasible tool for describing AR27 decoloration and its further degradation by the microbial consortium of *T. versicolor* and *P. putida*, this model will also work as a mathematical basis for designing novel bio-reaction systems than can operate with the same principle of the described consortium.

## 1 Introduction

The discharge of untreated wastewater from industries related to the use of synthetic dyes (textile, tannery, coatings, paper, cosmetics, pharmaceuticals, and dye manufacturing) are responsible for environmental pollution, either in terms of effluent content and/or volume [1,2]. The textile industry is the sector that contributes the most to water pollution by dyes discharges (54 %) [3]. Its effluents contain different types of dyes and other organic and inorganic pollutants [4]. It is estimated that about 280,000 or 300,000 tons per year of dyes that are being used worldwide in the textile industry will end up in the environment through wastewater discharges mainly due to a lack of fixation in the textile fibers during dyeing processes [5,6]. Different types of dyes are used in textile industry, the most widely used in dyeing processes are azo dyes (-N]N-) [7], accounting for 50–80 % of the organic dyes that are normally used [1,6,8]. Their concentration in textile wastewater is between 10 and 500 mg l^−1^ [9,10]; azo dyes also possess chemical and photolytic stability due to the aromatic chains within their molecular structure [2,10]. These pollutants are considered hazardous and persistent due to their bioaccumulation capacity, as well as the carcinogenic and mutagenic effects they can cause, also showing potential toxicity to the biosphere and anthroposphere [8,9,11], thus their treatment for removal and/or elimination from the environment, without causing secondary pollution, has become a priority [3].

Acid Red 27 (AR27), also known as Amaranth dye and Food Red 9, is a cationic monoazo dye chemically synthesized as a trisodium salt. Its coloration is dark red and it is widely used in paper processing, textiles, food, cosmetics, drinks, drugs and in the photographic industry. In the textile industry, it is widely used for dyeing natural and synthetic fibers, such as leather, wool and silk, where it presents a bright bluish red color from an acid bath. Due to its complex chemical structure, which includes an azo group and a conjugated benzene ring, AR27 is a persistent compound that is extremely recalcitrant to degradation. So, industrial effluents containing AR27 adversely affect humans and animals with effects that include respiratory problems, birth defects, allergies, tumors, genotoxicity, cytotoxicity, embryotoxicity, mutagenicity, carcinogenicity and endocrine disruption. Furthermore, wastewater discharges, containing AR27, into natural water bodies affect the viability and photosynthetic process of aquatic plants by reducing sunlight penetration and also deprives aquatic animals of the oxygen required for their vital functions [[12], [13], [14]].

Due to the nature of textile wastewater, their treatment can be carried out by means of physical, chemical and biological processes. Physical methods are generally simple and their operating principal is to remove the dye from the effluent and take it to a different matrix, without the dye changing its chemical structure [15]. They are noted for presenting ease of operation, simplicity in their design, low cost, no inhibitory effect due to the presence of toxic substances and have low chemical requirements [16,17]. Their main disadvantage is the production of large amounts of sludge, their limited applicability and the presence of toxic intermediate compounds due to low degradation efficiency [18]. Other factors affecting the applicability of these methods for textile wastewater treatment are high temperature, chemical oxygen demand (COD), pH and the presence of heavy metals [8]. The most common physical methods for textile wastewater treatment are ion exchange, membrane filtration and adsorption [8]. Chemical methods are the most commonly used for textile wastewater treatment. They are based on the degradation of dyes by chemical oxidation/reduction reactions through the addition of chemicals. These treatments present good removal and degradation efficiencies, are fast and compact, however, the main disadvantage is the high operating costs due to the use of specialized equipment, high energy requirements and the use of large amounts of chemicals [19,20]. Another challenge for these treatment methods is the generation of toxic metabolites and/or by-products formed during the process, which can increase the cost due to additional treatment equipment requirements [15]. Overall, chemical methods for the treatment of textile wastewater are chemical coagulation-flocculation, electrochemical processes, chemical oxidation processes and advanced oxidation processes [8,21]. Biological methods are considered more promising than chemical and physical methods due to advantages, such as: easier application, lower sludge generation, lower requirement of additional chemical agents, economic viability, environmental innocuity and wide applicability onto various types of dyes [2,7,8,[22], [23], [24]]. Biological methods take advantage of the metabolic potential of microorganisms, such as bacteria, fungi, yeasts, algae, enzymatic systems, etc., either in pure cultures or through microbial consortia, to transform the dye molecule into simpler and non-toxic compounds that can be eliminated by conventional processes [18,[23], [24], [25]]. Several species of fungi and bacteria are also capable of decolorizing and mineralizing dyes; therefore, they have been studied in the development of novel biological processes for textile wastewater treatment. These studies discuss the involvement of various extracellular reducing enzymes, such as: azoreductase and oxidative enzymes like laccase, tyrosinase and lignin manganese peroxidases; in the decoloration process [24,26,27]. The use of metabolically designed microbial consortia, for bioremediation of textile wastewater, offers significant advantages over the use of monocultures. The metabolic synergism between microorganisms allows the dye molecule to be transformed by stages, where by-products generated in the growth of one strain, during the initial decoloration, can be used as a substrate for growing another strain [6,28,29]. Microbial consortia can be classified into two types, defined consortia and undefined consortia. Undefined consortia are assumed as a natural population of degrading microorganisms spontaneously occurring in polluted environments. Defined consortia are those that are designed according to the characteristics of the pollutant to be biodegraded; generally, with a well-defined number of microbial strains that complement each other with their metabolic capabilities, in such a way that are selected and combined [30,31].

The kinetics of a bioreaction can be numerically described by mathematical modeling of the metabolic process. The development of a mathematical model should include the definition of the main parameters of the reaction system, their nature, and the effects they may have on it. Kinetic constants, associated to the biological process, are estimated by correlating the mathematical model to experimental data. Depending on the agreement of the modelled results, in comparison with the behavior of the experimental biological system, under different conditions, it can be predicted the performance of the biosystem using the mathematical model. Thereby, the mathematical model provides the basis for the design of the bioreaction system, as well as for the local optimization and the design of control strategies of the system [32,33]. Some researches have used the mathematical modeling approach to describe the biological process that follows the decoloration and/or degradation of textile industry dyes [[34], [35], [36], [37], [38], [39], [40], [41]], in which the importance of mathematical models for obtaining kinetic parameters governing dye decolorization processes and the subsequent application in the design, operation and optimization of bioreaction systems is highlighted.

The aim of this work is to propose a mathematical model, based on the Monod, Haldane-Andrews and Michaelis-Menten equations; for simulating the behavior, under different operating conditions, of a novel microbial consortium made up with *Trametes versicolor* and *Pseudomonas putida* during the decoloration and further degradation of AR27, including an initial proposal of local theoretical optimum conditions for the reaction process by a sensitivity analysis performed using the response surface method.

## 2 Materials and methods

### 2.1 Conceptual model

**Fig. 1:**
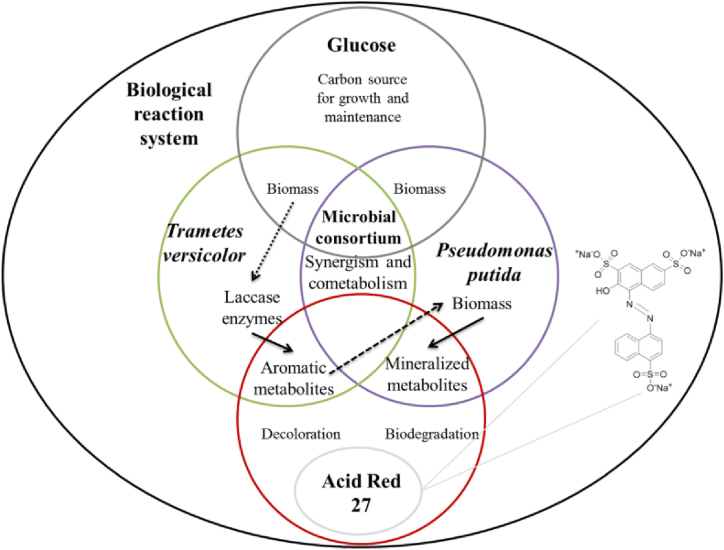
Conceptual model of a biological system formed by a novel microbial consortium of a fungus (*Trametes versicolor*) and a bacterium (*Pseudomonas putida*) for the decoloration of the azo dye acid red 27. The microorganisms interact metabolically during the biological process, which allows co-metabolism and symbiosis in the system. (For interpretation of the references to color in this figure legend, the reader is referred to the Web version of this article.)

The conceptual model theoretically describes the biological process of decoloration and breakdown of AR27 by metabolic interaction of the consortium of *T. versicolor* and *P. putida* [42]. Fig. 1 shows the conceptual model, and this has the following assumptions [43,44].

Image modified from Ref. [42]. The molecular structure of acid red dye 27 is shown [45].

(a) The consortium is composed of a strain of *T. versicolor*; a white-rot fungus widely studied in dye bioremediation and an organism capable of releasing laccase as its main extracellular enzyme [46].
b) A strain of *P. putida*, which is a Gramm negative bacterial specie commonly used for bioremediation studies of environmental contaminants, due to its ability to biodegrade aromatic compounds [47]. This organism might induce in the fungus an increased production of laccase enzymes, as a stress effect in mixed cultures.
c) Both microorganisms grow in the same culture medium, even though they may need different growth conditions. The growth medium is favorable for enzyme production.
d) For its growth, development, maintenance and enzyme production *T. versicolor* consumes glucose as its sole carbon source, while *P. putida* grows initially by consuming glucose and later its growth continues by consuming the aromatic compounds subsequently formed in the culture medium.
e) The laccase enzymes produced by *T. versicolor* are responsible for the decoloration of the growth medium. As a result of the initial decoloration, it is assumed that metabolites of aromatic nature are generated, such as benzene and naphthalene-derived compounds [48].
f) The aromatic metabolites of AR27, after the initial decoloration, are then consumed and degraded by *P. putida.* These metabolites are used as a carbon source for the bacterium and their disappearance allows a better growth of *T. versicolor.* Hence, it is inferred that a co-metabolic interaction between the fungus and the bacterium is set.
g) The biological system is analyzed in a batch mode.
h) The kinetic mathematical model is based on a general mass balance of a generic compound or specie, as described in Eq (1).

(1) $$\frac{dC\left(i\right)}{dt}=r\left(i\right)*C\left(i\right)$$

where “*C(i)*” represents the concentration of the species “*i*” involved in the system and “*r(i)*” represents the rate of change with respect to time “*t*”.

### 2.2 Mathematical model

This describes the process of decoloration and breakdown of AR27 by the consortium of *T. versicolor* and *P. putida*. Its development was based on the conceptual equations *I*, *II* and *III* that are shown, which describe the main stages guiding the biological process, as follows: (*I*) fungal growth, glucose uptake and enzymatic production, (*II*) AR27 breakdown and aromatics production, and (*III*) bacterial growth out of glucose/aromatics uptake [42].

(I) T.versicolorbiomass+Glucose→T.versicolorbiomass+Laccaseenzyme

(II) AR27dye+Laccaseenzyme→Aromaticcompounds

(III) $$P.\;putida\;biomass+Glucose+Aromatic\;compounds\to P.\;putida\;biomass+{CO}_{2}$$

### 2.3 Fungal growth

*T. versicolor* growth occurs in two stages: a short period of rapid growth followed by a period of deceleration and decay, as reported by Ikasari *et* Mitchell (2000) [49]. Exponential growth was described by Monod's Law of Velocity [50] where glucose was used as a growth substrate, maintenance and laccase production due to the affinity of the fungus for it [51]. Once the substrate is depleted, growth slows down and the decay phase begins. The Monod's Law of Velocity equation is substituted into Eq (1)., and Eq (2)., is obtained to describe fungal growth.

(2) $${rX}_{T}=\frac{{dX}_{T}}{dt}=\frac{\mu_{\max-T}*C_{glc}}{K_{sglcT}+C_{glc}}*X_{T}-K_{dT}X_{T}$$

where “${rX}_{T}$” is the growth rate $\left(g_{T}l^{-1}d^{-1}\right)$; “$\mu_{\max-T}$” is the maximum growth rate on the substrate, glucose $\left(d^{-1}\right)$; “$K_{sglcT}$” is the glucose saturation constant $\left(g_{glc}l^{-1}\right)$; “$C_{glc}$” the glucose concentration in the medium $\left(g_{glc}l^{-1}\right)$; “$X_{T}$” is the biomass concentration $\left(g_{T}l^{-1}\right)$ and “$K_{dT}$” is the cell decay constant $\left(d^{-1}\right)$.

### 2.4 Bacterial growth

In accordance with the conceptual model presented in section 2.1, the growth of *P. putida* is initially using glucose as a substrate and then continues its growth by consuming the aromatic compounds produced during the AR27 decoloration. Due to the fact that glucose is not a substrate that can inhibit bacterial growth, Monod's Speed Law was used to describe the first phase of *P. putida* growth [52]. As a second phase of bacterial growth, it was assumed that aromatic compounds may exhibit inhibition due to their toxicity, so bacterial growth can be described by the Haldane-Andrews' equation [52,53]. Out of the inhibitory microbial growth models, the Haldane-Andrews' model was selected because of its mathematical simplicity and wide acceptance. Eq (3)., describes the bacterial growth.

(3) rXP=dXPdt=(μmax−P*CglcKsPglc+Cglc+μmax−aP*CaroKsaro+Caro+(Caro2Ki))*XP−KdPXP

where “${rX}_{P}$” is the growth rate $\left(g_{P}l^{-1}d^{-1}\right)$; “$\mu_{\max-P}$” is the maximum growth rate on the substrate, glucose $\left(d^{-1}\right)$; “$K_{sPglc}$” is the glucose saturation constant $\left(g_{glc}l^{-1}\right)$; “$C_{glc}$” the glucose concentration in the medium $\left(g_{glc}l^{-1}\right)$; “$\mu_{\max-aP}$” is the maximum growth rate on the substrate, aromatic compounds $\left(d^{-1}\right)$; “$K_{saro}$” is the saturation constant of aromatic compounds $\left(g_{aro}l^{-1}\right)$; “$C_{aro}$” the concentration of aromatic compounds $\left(g_{aro}l^{-1}\right)$; “$K_{i}$” is the inhibition constant of aromatic compounds $\left(g_{aro}l^{-1}\right)$; “$X_{P}$” is the biomass concentration $\left(g_{P}l^{-1}\right)$ and “$K_{dP}$” is the cell decay constant $\left(d^{-1}\right)$.

### 2.5 Substrate consumption

Glucose consumption as the main substrate for microbial growth was described by the Monod equation, which represents the substrate consumption for two purposes of cell viability [51,53]. Eq (4)., describes the relationship between glucose consumption and biomass generation of both microorganisms and, specifically, for *T. versicolor* in the production of laccase enzymes.

(4) $${-r}_{glc}=-\frac{dglc}{dt}=\left[\frac{\mu_{fungus}*X_{T}}{Y_{T-glc}}+\left(\frac{X_{T}*K_{enz}}{Y_{enz-glc}}\right)\right]+\left[\frac{X_{P}*\mu_{bacteria}}{Y_{P-glc}}\right]$$

with

(5) $$\mu_{fungus}=\frac{\mu_{\max-T}*C_{glc}}{K_{sglcT}+C_{glc}}$$

and

(6) μbacteria=μmax−P*CglcKsPglc+Cglc+μmax−aP*CaroKsaro+Caro+(Caro2Ki)

where “$-r_{glc}$” is the rate of glucose consumption $\left(g_{glc}l^{-1}d^{-1}\right)$; “$\mu_{fungus}$” (in Eq (5).) is the Monod's term describing growth for *T. versicolor* $\left(d^{-1}\right)$; “$Y_{T-glc}$” is the biomass yield of *T. versicolor* from glucose $\left(g_{T}g_{glc}^{-1}\right)$; “$X_{T}$” is the biomass concentration of *T. versicolor* $\left(g_{T}l^{-1}\right)$; “$K_{enz}$” is the enzyme production constant $\left(U_{enz}g_{T}^{-1}d^{-1}\right)$; “$Y_{enz-glc}$” is the laccase enzyme yield from glucose $\left(U_{enz}g_{glc}^{-1}\right)$; “$\mu_{bacteria}$” (in Eq (6).) is the Monod's term describing growth for *P. putida* $\left(d^{-1}\right)$; “$Y_{P-glc}$” is the biomass yield of *P. putida* from glucose $\left(g_{P}g_{glc}^{-1}\right)$ and “$X_{P}$” is the biomass concentration of *P. putida* $\left(g_{P}l^{-1}\right)$.

### 2.6 Enzyme production

Laccase production, by *T. versicolor*, is due to survival issues when it interacts with oxidizing agents or organisms that may cause risk [54]. In this case, the laccase production may be a response to nutrient limitations in the environment and the stress caused by the presence of *P. putida* and the dye AR27. Eq (7)., describes the laccase enzyme production and decay.

(7) $$r_{enz}=\frac{dC_{enz}}{dt}=K_{enz}X_{T}e^{\mu_{fungus}t}-\left[\frac{1}{Y_{col-enz}}\left(\frac{V_{\max}{*C}_{col}}{K_{{ME}_{Col}}+C_{col}}\right)*C_{enz}*V_{t}+K_{d}C_{enz}\right]$$

Where “$r_{enz}$” is the rate of laccase enzyme production $\left(U_{enz}l^{-1}d^{-1}\right)$; “$K_{enz}$” is the enzyme production constant by *T. versicolor* $\left(U_{enz}g_{T}^{-1}d^{-1}\right)$; “$\mu_{fungus}$” (from Equation (5)) is the Monod's term describing cell growth of *T. versicolor* $\left(d^{-1}\right)$; “$X_{T}$” is the biomass concentration of *T. versicolor* $\left(g_{T}l^{-1}\right)$; “t” is the time (d); “$Y_{col-enz}$” is the enzyme ratio coefficient with respect to the degraded dye $\left(g_{col}U_{enz}^{-1}\right)$; “$V_{\max}$” is the maximum rate of decoloration of AR27 by laccase $\left(g_{col}U_{enz}^{-1}l^{-1}d^{-1}\right)$; “$K_{MEcol}$” is the Michaelis-Menten constant for the dye-laccase ratio $\left(g_{col}l^{-1}\right)$; “$C_{col}$” is the concentration of AR27 in the medium $\left(g_{col}l^{-1}\right)$; “$V_{t}$” is the total volume of the reaction system (l); “$K_{d}$” is the laccase enzyme decay constant $\left(d^{-1}\right)$ and “$C_{enz}$” is the laccase enzyme concentration $\left(U_{enz}l^{-1}\right)$.

### 2.7 AR27 decoloration

To date, many kinetic studies of laccase enzyme-catalyzed reactions for the decoloration of textile dyes have applied the Michaelis-Menten kinetic model [55]. This model was developed under the assumptions that the free enzyme combines with the substrate to form the “enzyme-substrate” complex, which later separates into product and enzyme. It is currently a widely used model. According to Cristóvão et al. (2008) [40], the degradation kinetics catalyzed by the laccase enzyme for a single dye can be described by the Michaelis-Menten equation. AR27 decoloration is a reaction catalyzed by the laccase enzyme produced by *T. versicolor* and can be described through the Michaelis-Menten kinetic model [26,36,38,40]. The mathematical expression describing dye decoloration is given by Eq (8).

(8) $$-r_{col}=-\frac{d_{col}}{dt}=\left(\frac{V_{\max}*C_{col}}{K_{{ME}_{Col}}+C_{col}}\right)*C_{enz}*V_{t}$$

Where “${-r}_{col}$” is the degradation rate of AR27 $\left(g_{col}l^{-1}d^{-1}\right)$; “$V_{\max}$” is the maximum rate of AR27 decoloration by laccase $\left(g_{col}U_{enz}^{-1}l^{-1}d^{-1}\right)$; “$K_{MEcol}$” is the Michaelis-Menten constant for the dye-laccase ratio $\left(g_{col}l^{-1}\right)$; “$C_{col}$” is the concentration of AR27 in the medium $\left(g_{col}l^{-1}\right)$; “$V_{t}$” is the total volume of the reaction system (l) and “$C_{enz}$” is the laccase enzyme concentration $\left(U_{enz}l^{-1}\right)$.

### 2.8 Metabolites breakdown

The production and consumption of aromatic compounds in the biological system is a result of AR27 decoloration initiated by the fungal laccase, and further aromatics degradation by *P. putida*, which can be described by Eq (9).

(9) $$r_{aro}=\frac{daro}{dt}=a*K_{dec}*C_{col}-\left[\frac{\mu_{bacteria}*X_{P}}{Y_{P-aro}}\right]$$

Where “$r_{aro}$” is the rate of uptake of aromatic compounds $\left(g_{aro}l^{-1}d^{-1}\right)$; “a” is the stoichiometric coefficient of aromatic compounds from the dye $\left(g_{aro}g_{col}^{-1}\right)$; “$K_{dec}$” is the degradation constant of the dye $\left(d^{-1}\right)$; “$C_{col}$” is the concentration of AR27 in the medium $\left(g_{col}l^{-1}\right)$; “$\mu_{bacteria}$” (from Eq (6).) is the Monod's term describing cell growth of *P. putida* $\left(d^{-1}\right)$; “$Y_{P-aro}$” is the biomass yield of *P. putida* from aromatic compounds $\left(g_{P}g_{aro}^{-1}\right)$ and “$X_{P}$” is the biomass concentration of *P. putida* $\left(g_{P}l^{-1}\right)$.

### 2.9 Solution of the mathematical model

The model (described from Eq (2)., Eq (3)., Eq (4)., Eq (7)., Eq (8). And Eq (9).) was solved in Microsoft Excel® 2010 by applying the 4th order Runge-Kutta integration method [56].

### 2.10 Mathematical model parameters

The kinetic parameters for *T. versicolor* and for *P. putida* used for the solution of the mathematical model are presented in Table 1. These were obtained from literature and internal experiments, previously reported in private communications of the contributors to this work [42].

**Table 1:** Kinetic parameters for *T. versicolor* and *P. putida* during mathematical modeling of AR27 decoloration. Taken from Ref. [42].

Parameter	Symbol	Value	Units	Reference
Kinetic parameters for *Trametes versicolor*				
Yield of laccase from glucose	Y_enz-glc_	48.63	Ug^−1^	[57]
Maximum growth rate in glucose	μ_max-T_	0.87	d^−1^	[50]
Glucose saturation constant	K_sglcT_	4.21	gl^−1^	
Cell decay constant	K_dT_	0.09	d^−1^	
Biomass yield from glucose	Y_T-glc_	0.88	gg^−1^	Experimental parameter of this study [43]
Maximum decoloration rate	V_max_	0.03	gU^−1^l^−1^d^−1^	
Michaelis-Menten constant for dye-laccase ratio	K_MEcol_	1.003	gl^−1^	
Laccase enzyme production constant	K_enz_	1932	Ug^−1^d^−1^	[41]
Enzyme decay constant	K_d_	0.67	d^−1^	
AR27 degradation constant	K_dec_	0.13	d^−1^	[58]
Ratio of enzyme to degraded dye ratio	Y_col-enz_	0.87	g_col_U_enz_^−1^	Estimated value [44]
**Kinetic parameters for *Pseudomonas putida***				
Maximum growth rate in glucose	μ_max-P_	0.36	d^−1^	Experimental parameter of this study [43]
Glucose saturation constant	K_sglcP_	3.68	gl^−1^	
Biomass yield from glucose	Y_P-glc_	0.55	gg^−1^	
Maximum growth rate in aromatic compounds	μ_max-aP_	0.31	d^−1^	[59]
Saturation constant in aromatic compounds	K_saro_	0.03	gl^−1^	
Aromatic compound inhibition constant	K_i_	0.26	gl^−1^	
Cell decay constant	K_dP_	0.06	d^−1^	
Biomass yield from aromatic compounds	Y_P-aro_	0.59	gg^−1^	
Stoichiometric coefficient of aromatic compounds from AR27	*a*	2.35	gg^−1^	Estimated value [44]

### 2.11 Simulation scenarios

The solution of the model included simulation of three different scenarios, in which the initial conditions were modified for analyzing the stability of the model. The initial biomass concentration was maintained in all three scenarios, being 0.025 g l^-1^ for *T. versicolor* and 0.0065 g l^-1^ for *P. putida* [43] setting at three levels the initial glucose and dye concentrations, along with the moment in time for inoculation of *P. putida*. The values for each level were labeled as: low level (***l***), medium level (***m***) and high level (***h***); being: three initial glucose concentrations (10, 14 and 20 g l^-1^); three initial AR27 concentrations (0.2, 0.5 and 1.0 g l^-1^); and three bacterial inoculation times (1, 2 and 3 days) that were evaluated. The scenarios were identified by combining symbols and letters to differentiate, with (**α**) as the symbol to identify glucose concentration; (**β**) for AR27 dye concentration; and (**γ**) for bacterial inoculation time; followed by the corresponding levels.

### 2.12 Sensitivity analysis by Response Surface Method (RSM)

In order to study the independent and combined effects of operational conditions on respond variables of interest, the RSM method was chosen because of its statistical significance and accuracy when generating polynomial models out of kinetic data. RSM was a useful alternative for analysing the influence of three independent variables (see Table 2), on the response of three dependent variables to be optimized upon the minimum time (in days) for: full decoloration of AR27 (R_1_); maximum production of aromatic metabolites (R_2_); and full depletion of aromatic metabolites (R_3_); which were studied using RSM with a full factorial design 3^3^. The 27 results obtained from the simulation were submitted to a sensitivity analysis by means of multivariable regression in which the corresponding mathematical models were developed using Microsoft Excel® 2010. The subsequent response surface graphs were made with SigmaPlot 14.0.

**Table 2:** Levels of the independent variables for the full factorial experimental design 3^3^.

Factor	Symbol	Units	Low level (l)	Medium level (m)	High level (h)
Glucose concentration	Α	gl^−1^	10	14	20
Dye concentration	Β	gl^−1^	0.2	0.5	1.0
*P. putida* inoculation	Γ	day	1	2	3

## 3 Results and discussion

### 3.1 Fungal growth, substrate consumption and enzyme production

The biological process of decoloration and further degradation of AR27 by the consortium of *T. versicolor* and *P. putida* follows two stages, the first focused on dye decoloration and the second focused on metabolite breakdown. The first stage starts with the growth of *T. versicolor* for the production of laccase, which is associated with biomass concentration [60]; in that sense, enzyme production will be higher as long as the fungal biomass concentration increases.

**Fig. 2:**
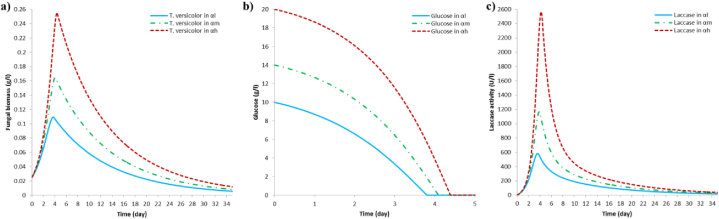
Simulation results in terms of *T. versicolor* growth, glucose consumed and *T. versicolor* laccase production. (a) *T. versicolor* growth in **α*l***, **α*m*** and **α*h*** scenarios; (b) glucose consumption in **α*l*** (10 g l^-1^), **α*m*** (14 g l^-1^) and **α*h*** (20 g l^-1^) scenarios by *T. versicolor* and *P. putida* consortium during decoloration of AR27 and (c) laccase production by *T. versicolor* in scenarios involving **α*l***, **α*m*** and **α*h*** during decoloration of AR27.

In Table 3 are presented the results of the whole simulations for each scenario in terms of the maximum fungal growth, the complete glucose consumption and the maximum laccase activity. It is to be highlighted that the inoculation time of *P. putida* (**γ**) and the initial AR27 concentration (**β**) seem to have no influence on the maximum growth of the fungus neither on the glucose depletion time. There is no change in fungal growth results in scenarios with the same glucose concentration, such as (**α*l*/β*l-m-h*/γ*l-m-h***), (**α*m*/β*l-m-h*/γ*l-m-h***) and (**α*h*/β*l-m-h*/γ*l-m-h***). These results were as expected because the dye is not a carbon source for the fungus [43,44], so that fungal growth does not depend on the initial AR27 concentration. Fig. 2a shows the simulations of fungal growth in each scenario. In Fig. 2b it is presented the glucose depletion curves during microbial growth in (**α*l*/β*l-m-h*/γ*l-m-h***), (**α*m*/β*l-m-h*/γ*l-m-h***) and (**α*h*/β*l-m-h*/γ*l-m-h***) scenarios. It is to be highlighted that when *T. versicolor* reaches its maximum growth, 97.39 % of the glucose has been consumed in scenario **α*l***; 98.78 % of the glucose in scenario **α*m*** and 99.43 % of the glucose in scenario **α*h***. A remarkable issue is the fact that there is no change in the maximum growth of the fungus or in the day of glucose depletion when the inoculation time of the bacteria is changed. This phenomenon may be due to the fact that *P. putida* grows preferentially consuming decoloration metabolites upon glucose [61]. This particular case will be discussed in more detail in section 3.5.

**Table 3:** Results of simulations of the decoloration and degradation process of AR27 by the microbial consortium of *T. versicolor* and *P. putida*.

Run	Scenario	A factor	B factor	C factor	*Results*						
		Glucose concentration	AR27 concentration	*P. putida* inoculation	*Maximum T. versicolor* growth		*Maximum laccase activity*		*Complete glucose consumption*	*Maximum P. putida* growth	
		gl^−1^	gl^−1^	day	*gl*^*−*^*^1^*	*day*	*Ul*^*−*^*^1^*	*day*	*Day*	*gl*^*−*^*^1^*	*day*
1	**(α*l/*β*l/*γ*l*)**	**10**	**0.2**	**1**	0.11	3.70	583.43	3.58	3.825	0.03	11.53
2	**(α*l/*β*l/*γ*m*)**	**10**	**0.2**	**2**	0.11	3.70	583.89	3.58	3.825	0.03	14.03
3	**(α*l/*β*l/*γ*h*)**	**10**	**0.2**	**3**	0.11	3.73	584.18	3.58	3.825	0.02	16.48
4	**(α*l/*β*m/*γ*l*)**	**10**	**0.5**	**1**	0.11	3.70	583.38	3.58	3.825	0.07	16.05
5	**(α*l/*β*m/*γ*m*)**	**10**	**0.5**	**2**	0.11	3.70	583.84	3.58	3.825	0.07	18.63
6	**(α*l/*β*m/*γ*h*)**	**10**	**0.5**	**3**	0.11	3.73	584.12	3.58	3.825	0.07	21.05
7	**(α*l/*β*a/*γ*l*)**	**10**	**1**	**1**	0.11	3.70	583.47	3.58	3.825	0.15	25.00
8	**(α*l/*β*a/*γ*m*)**	**10**	**1**	**2**	0.11	3.73	583.80	3.58	3.825	0.15	28.23
9	**(α*l/*β*a/*γ*h*)**	**10**	**1**	**3**	0.11	3.73	584.01	3.58	3.825	0.15	31.15
10	**(α*m/*β*l/*γ*l*)**	**14**	**0.2**	**1**	0.16	4.05	1167.34	3.88	4.10	0.03	10.38
11	**(α*m/*β*l/*γ*m*)**	**14**	**0.2**	**2**	0.16	4.05	1168.23	3.88	4.10	0.03	13.00
12	**(α*m/*β*l/*γ*h*)**	**14**	**0.2**	**3**	0.16	4.05	1168.81	3.88	4.10	0.02	15.73
13	**(α*m/*β*m/*γ*l*)**	**14**	**0.5**	**1**	0.16	4.05	1167.31	3.88	4.10	0.07	14.85
14	**(α*m/*β*m/*γ*m*)**	**14**	**0.5**	**2**	0.16	4.05	1168.20	3.88	4.10	0.07	17.60
15	**(α*m/*β*m/*γ*h*)**	**14**	**0.5**	**3**	0.16	4.05	1168.77	3.88	4.10	0.06	20.30
16	**(α*m/*β*h/*γ*l*)**	**14**	**1**	**1**	0.16	4.05	1167.66	3.88	4.10	0.15	23.23
17	**(α*m/*β*h/*γ*m*)**	**14**	**1**	**2**	0.16	4.05	1168.30	3.88	4.10	0.14	26.65
18	**(α*m/*β*h/*γ*h*)**	**14**	**1**	**3**	0.16	4.05	1168.72	3.88	4.10	0.14	29.95
19	**(α*h/*β*l/*γ*l*)**	**20**	**0.2**	**1**	0.26	4.38	2562.70	4.20	4.40	0.03	9.18
20	**(α*h/*β*l/*γ*m*)**	**20**	**0.2**	**2**	0.26	4.38	2564.50	4.20	4.40	0.03	11.80
21	**(α*h/*β*l/*γ*h*)**	**20**	**0.2**	**3**	0.26	4.38	2565.70	4.20	4.40	0.02	14.70
22	**(α*h/*β*m/*γ*l*)**	**20**	**0.5**	**1**	0.26	4.38	2562.63	4.20	4.40	0.07	13.60
23	**(α*h/*β*m/*γ*m*)**	**20**	**0.5**	**2**	0.26	4.38	2564.48	4.20	4.40	0.06	16.48
24	**(α*h/*β*m/*γ*h*)**	**20**	**0.5**	**3**	0.26	4.38	2565.68	4.20	4.40	0.06	19.40
25	**(α*h/*β*h/*γ*l*)**	**20**	**1**	**1**	0.26	4.38	2563.55	4.20	4.40	0.14	21.48
26	**(α*h/*β*h/*γ*m*)**	**20**	**1**	**2**	0.26	4.38	2564.88	4.20	4.40	0.14	25.08
27	**(α*h/*β*h/*γ*h*)**	**20**	**1**	**3**	0.26	4.38	2565.76	4.20	4.40	0.14	28.65

(**α**) Symbol to identify glucose concentration.

(**β**) Symbol to identify AR27 dye concentration.

(**γ**) Symbol to identify bacterial inoculation time.

(***l)*** Low level.

(***m***) Medium level.

(***h***) High level.

Initial experimental assays (not published) showed that there is no inhibition halo between the two microorganisms when they grow in the same culture medium, however if both microorganisms are inoculated at the same time, *P. putida* inhibits the growth of *T. versicolor*, so it was decided to implement a growth technique where the survival of both species was guaranteed: the fungus is initially grown as a pure culture long enough to begin AR27 decoloration and the bacterium is inoculated 72 h later [43,44]. Another preliminary assay was to qualitatively determine the ability of *T. versicolor* and *P. putida* to decolorize AR27. The results showed that *T. versicolor* has the ability to decolorize the dye, however, it cannot use it as a carbon source and, without the presence of glucose the fungus does not grow. On the other hand, *P. putida* can grow in the presence of the dye but does not have the ability to decolorize [44]. The next step in the experimental assays was to compare the growth of the species as pure cultures and in consortium, which resulted in a higher fungal growth rate in consortium in comparison to growth as a pure culture and growth in consortium was higher with the presence of the dye than without the presence of AR27, determined at 158 h [43]. Regarding glucose consumption, the results showed that *T. versicolor* consumed 75 % of the substrate at 200 h with and without the presence of AR27, while in consortium with *P. putida* the same percentage of consumption was also achieved at 200 h without the presence of the dye and was reduced to 123 h with the presence of the dye [43]. These results indicate that the presence of AR27 in the consortium induces glucose consumption because when faced with the need to remove the contaminant (AR27) from the medium, both microorganisms use more resources from the environment for enzyme production and the microbial interaction promotes mutual growth [43].

Fungal growth is stimulated with an enriched culture media or using chemically synthesized compounds. Atilano-Camino et al. (2020) [62] carried out a comparative study to improve microbial growth and laccase production of *T. versicolor* using lignocellulosic residues (agave bagasse, coconut fibers and wheat bran) as co-substrates. Biological consortia are a class of natural inducers [43,63,64] and, in that sense, it can be considered that the presence of *P. putida*, besides working as a microorganism degrading aromatic compounds, also acts as an inducing agent for cell growth of *T. versicolor* and, therefore, increases laccase production.

The use of a microbial consortium can provide a better colonization of the substrate, a higher production of relevant enzymes and a higher resistance to contamination by other microorganisms [6,65]. In that context, two factors are important for the success of a bioprocess: nutritional factors and species compatibility, which is defined by the existing metabolic interactions between species. Concerning the former, the carbon source, the nature of nitrogen and the ratio between these two elements (C/N) play an important role for the metabolism of microorganisms cultivated in a consortium [66,67]. Namely, the C:N ratio should be greater than 10 [48] to enhance the production of laccases.

Glucose is a carbon source easily assimilated by most microorganisms [34,60,63,68] and its presence is necessary for fungal growth and subsequent production of laccases. For the model design, glucose was the main carbon source for *T. versicolor* and *P. putida*.

The biodegradation of synthetic dyes by fungi can be carried out by two processes: through the catalytic action of extracellular enzymes, produced by the microorganism and/or by the adsorption of the dye in the biomass followed by intracellular enzymatic degradation [69,70]. The mathematical model developed in this work, considers the AR27 decoloration through an enzymatic reaction of laccase as it was reported to be the most useful extracellular ligninolytic enzyme from *T. versicolor* in dye decoloration research [66,71].

Laccase is a group of enzymes belonging to the blue oxidoreductases that contain copper atoms inside their catalytic centers capable of using oxygen as a final electron acceptor. Laccase catalyzes the oxidation of a variety of organic compounds, such as phenols and aromatic amines, as well as non-phenolic compounds, so their use for bioremediating environmental pollutants is increasing [72,73]. Fungal laccases are more attractive for applications in bioremediation as they are not specific to a substrate and then have a high potential for oxidizing a wide range of pollutants [74]. Several studies reported laccase activity for decolorating textile dyes, such as: the commercial tannery dye, Dycem black [75]; acid orange 7, acid blue 74, reactive red 2 and reactive black 5 [71]; acid red 27 [76]; tartrazine and Allura red [66]; orange G, Congo red, direct blue 15, rose Bengal and direct yellow 27 [77]. Generation of laccase enzymes is associated with fungal growth, and this phenomenon can be observed in Fig. 2c, in which simulations show that, for the **α*l*** scenario a maximum laccase activity of 583.79 Ul^−1^ is achieved at 3.575 days; meanwhile the **α*m*** scenario calculates a maximum laccase activity of 1168.15 Ul^−1^ at 3.875 days, and for the **α*h*** scenario the maximum laccase activity is 2564.43 Ul^−1^ at 4.20 days.

Results obtained from the simulations are in the range of experimental values reported in the literature. In 2020, M. Tisma et al. [9] obtained laccase activity ranges of *T. versicolor* cultures of 270–306 Ul^−1^ at 7 days of culture using eggshells as an inducing agent during the degradation of aniline-based dyes. Meanwhile, Dauda *et* Erkurt (2020) [78] observed a maximum laccase activity of 15,358.28 Ul^−1^ at 14 days of *T. versicolor* culture on pampas grass (*Cortaderia selloana*) during the degradation of Reactive Blue 19. M Göksel (2019) [79] studied the degradation of six dyes (Remazol Red, Remazol Blue, Remazol Violet, Remazol Yellow, Remazol Orange and Remazol Black) through the production of laccases by *T. versicolor*. In his study, he used phenol as an inducer and obtained a maximum activity, after 6 days of culture, of 400 Ul^−1^. Finally, Stoilova *et* Krastanov (2008) [80] conducted a study of laccase overproduction (97,600 Ug^−1^ dry weight) with a mixed culture of *T. versicolor* and *Aspergillus niger*. The production of laccases depends on the culture conditions and the composition of the nutrient medium. The level of laccase production can be enhanced through the use of mediators and inducers. Mediators are low molecular weight molecules that are easy to oxidize by laccases and, once oxidized, these transport electrons to form more complex molecules [81]. Inducers, on the other hand, are molecules or organisms that affect the metabolism of the laccase-producing organism, by stressing it [80,82] or by influencing it at the genetic level, allowing an over-expression of laccase activity. Microbial consortia fungus/fungus [66,83]; fungus/bacteria [10,63], etc., are considered as a type of biological inducers due to the result of phenomena related to morphological changes and growth patterns of the species forming the consortium, diversification of laccase isoforms and generation of secondary metabolites. Kuhar et al. (2015) [83] found that the laccase activity of the fungal consortium of *T. versicolor* and *Ganoderma lucidum* was nine times higher than that recorded in individual cultures of each species, as well as the enzyme isoform; a fact that suggests that the effects of interspecific interactions between white-rot fungi and other microorganisms enhance laccase activity.

For this study, initial experimental results showed that in pure cultures of *T. versicolor* the maximum laccase activity was 220 Ul^−1^ achieved at 128 h, while in consortium, the maximum laccase activity was achieved at 168 h and was 851.8 Ul^−1^ [43]. This shows that microbial consortia act as biological inducers.

### 3.2 AR27 decoloration

The second stage of the biological process was simulated focusing on the enzymatic decoloration of AR27 followed by the production and breakdown of aromatic metabolites by action of *P. putida*.

The process of bioremediation of synthetic dyes by microbial consortia can follow two processes. The first is decoloration, which refers to the removal or transformation of the chromophore group (-N]N-) of the dye by breaking the double bond between nitrogen atoms [48,84,85]. However, this process does not guarantee the innocuity of the generated by-products, as they may present toxic characteristics more hazardous than those of the initial dye [22,86].

On the other hand, biodegradation is the process by which microorganisms transform complex chemical compounds into simpler molecules through their metabolism [87,88]. These complex molecules can serve as a carbon source for microorganisms and enable their cell growth and maintenance [29].

Based on the work of Gavril *et* Hodson (2007) [48], the degradation kinetics of the dye AR27 by T*. versicolor* follows the next stages: (a) dye decoloration starts with the formation of the phenoxy radical from the hydroxyl of the aromatic ring; (b) After electron redistribution, AR27 carbon structure is reduced to substituted naphthalene or benzene rings; (c) The azo bond can be transformed into (-NH_2_), (=NH) radicals attached to an aromatic ring; or released as (N_2_); (d) Sulfonic groups are gradually removed from the AR27 carbon structure; (e) An additional group, due to the conjugation of (C]C) and (C]O) bonds, could belong to a carbonyl group of a carboxylic acid attached to an aromatic ring. Due to these degradation kinetics, the decoloration compounds that can be formed are benzene (classified by the EPA as a known human carcinogen for all routes of exposure, causing blood disorders and leukemia [89]); toluene (toxic compound that mainly affects the central nervous system in humans and animals [90]), aniline (chemical recognized as a priority pollutant, persistent and bio accumulative that exhibits toxic effects to humans and has genotoxicity characteristics in plants. It is toxic, a probable Group B2 carcinogen and mutagenic in humans and animals [91,92]); benzoic acid (chemical of low toxicity in humans [93]); and naphthalene (compound classified by the EPA as a possible Group C human carcinogen, presenting minor health risks such as skin irritation and nausea to red blood cell breakdown, severe hemolysis and methemoglobinemia. It is also toxic to aquatic environments [94,95]). Considering the hazards of potential decoloration compounds, it is essential to eliminate their presence from the environment. Therefore, the contribution of *P. putida* in the consortium is required, because given its metabolic capabilities, it is expected that the intermediate compounds of the AR27 decoloration will be biodegraded and may even be mineralized.

In this work, it is assumed that the full biodegradation process is carried out by *P. putida*, which is a bacterial specie that holds the ability of degrading organic compounds with aromatic nature [47,96] by established metabolic pathways [97]. Bioremediation studies of AR27 have been conducted in which decoloration metabolites are reported, for example, Gomi et al. (2011) [98] studied the decoloration and biodegradation of AR27 by the fungus *Bjerkandera adusta* Dec. 1. They identified 1-aminonaphthalene-2,3,6-triol, 4-(hydroxyamino) naphthalene-1-ol and 2-hydroxy-3-[2-(4-sulfophenyl) hydrazinyl] benzenesulfonic acid as intermediate metabolites in a first 3-day decoloration step. After 10 days, they were able to identify 4-nitrophenol, phenol, 2-hydroxy-3-nitrobenzenesulfonic acid, 4-nitrobenzenesulfonic acid and 3,4′-disulfonyl azo benzene, suggesting that no aromatic amines were created. In a further study by Chhabra et al. (2015) [76], laccase produced by *Cyathus bulleri* was immobilized on polyvinyl alcohol beads cross-linked with nitrate or boric acid and through the operation of a packed column bioreactor the bioreaction was carried out. The metabolites identified were 4-((2-oxo-3, 6-disulfo-2,3-dihydronaphthalene-1-yl) diazenyl) naphthalene-1-sulfonate, 4-diazenylnaphthalene-1-sulfonate, naphthalene-1-sulfonate and 3,4-dioxo-7-sulfo-2,3,4,4a-tetrahydronaphthalene-2-sulfonate. In the study by Adnan et al. (2016) [99], 1,4-naphthalenediol, 1,2-dihydroxynaphthalene and coumarin were identified when investigating the biodegradation of AR27 by *Armillaria* sp. F022 in liquid medium. Based on our review, no studies of AR27 biodegradation by fungal/bacterial microbial consortia have been found, this is the first to describe the metabolic interactions of two different species during AR27 biodegradation.

The mathematical model, developed in this work, considers a first order AR27 degradation kinetics [58] and only the catalytic activity of the laccase is included as the main responsible for the decoloration. The kinetic parameters used for this enzymatic reaction are, according to initial experimental results (not published) [43], a maximum reaction rate (V_max_) of 0.032 g_col_U^−1^l^−1^d^−1^ and a Michaelis-Menten constant (*K*_*M*_) of 1.003 g_col_l^−1^. The values of these two kinetic constants suggest a high affinity of the enzyme for the dye, thus it is inferred that the proposed microbial consortium of *T. versicolor* and *P. putida* can tolerate high dye concentrations in textile wastewater. The results of simulations, in which three initial dye concentrations (0.2, 0.5 and 1.0 g l^-1^) were evaluated for each scenario, show that even a low laccase activity is required to initially break down the dye molecule and can catalyze high dye concentrations in similar times.

**Fig. 3:**
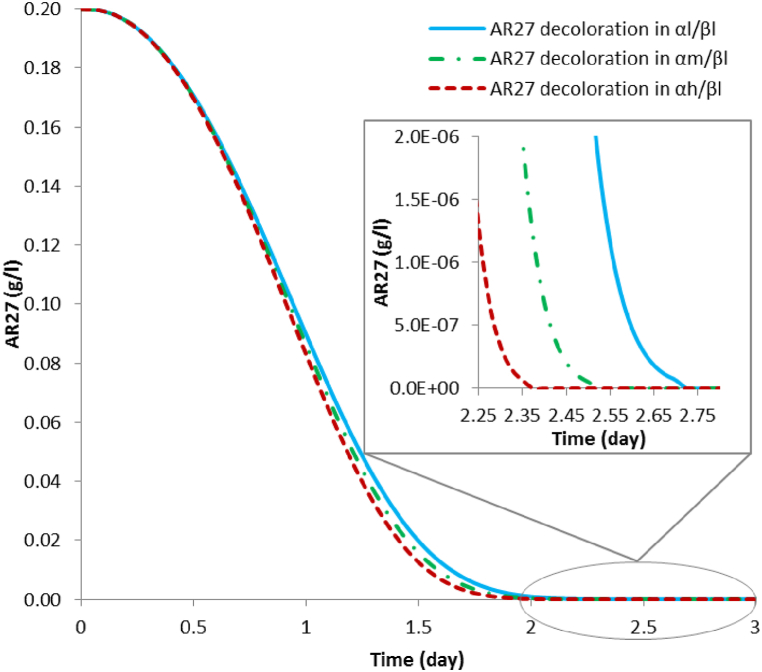
Simulation results of full decoloration of 0.2 g l^-1^ of AR27 by laccase produced with a *T. versicolor* and *P. putida* consortium in scenarios **α*l*/β*l***; **α*m/*β*l***; **α*h/*β*l***.

The results of AR27 full decoloration times (shown in Table 4), considering the scenarios ranging **α** and **β**, are the same disregarding the inoculation time of *P. putida* (**γ**), however, they differ according to the initial concentration of glucose (**α**) and the initial concentration of AR27 (**β**), such that they increase as the AR27 concentration increases and decrease as the initial glucose concentration increases. The decoloration curves for the scenarios involving (**β*l*)** are shown in Fig. 3. In the **α*l*/β*l*** scenarios, the complete decoloration of AR27 occurs with an associated laccase activity of 410.92 Ul^−1^; for the **α*m*/β*l*** scenarios, the complete decoloration of AR27 occurs with an associated laccase activity of 479.51 Ul^−1^; and for the **α*h*/β*l*** scenario, the complete decoloration of AR27 occurs with an associated laccase activity of 517.93 Ul^−1^.

**Table 4:** Simulation results of predicted times (days) for full AR27 decoloration by laccase from *T. versicolor*, along with the maximum production and full depletion of aromatic metabolites by *P. putida.*

Full AR27 decoloration (days)				
Initial glucose concentration	Initial AR27 concentration			Inoculation time of *P. Putida*
	β*l* (0.2 g l^-1^)	β*m* (0.5 g l^-1^)	β*h* (1.0 g l^-1^)	
**α*l* (10 g l**^**-1**^**)**	2.73	2.78	2.83	**γ*l* (1 day)**
	2.73	2.78	2.83	**γ*m* (2 day)**
	2.73	2.78	2.83	**γ*h* (3 day)**
**α*m* (14 g l**^**-1**^**)**	2.53	2.55	2.60	**γ*l* (1 day)**
	2.53	2.55	2.60	**γ*m* (2 day)**
	2.53	2.55	2.60	**γ*h* (3 day)**
**α*h* (20 g l**^**-1**^**)**	2.33	2.40	2.43	**γ*l* (1 day)**
	2.33	2.40	2.43	**γ*m* (2 day)**
	2.33	2.40	2.43	**γ*h* (3 day)**
**Maximum aromatic metabolites production (days)**				
**α*l* (10 g l**^**-1**^**)**	1.70	1.90	2.08	**γ*l* (1 day)**
	2.03	2.03	2.13	**γ*m* (2 day)**
	2.80	2.80	2.85	**γ*h* (3 day)**
**α*m* (14 g l**^**-1**^**)**	1.63	1.80	1.98	**γ*l* (1 day)**
	2.03	2.03	2.03	**γ*m* (2 day)**
	2.55	2.58	2.63	**γ*h* (3 day)**
**α*h* (20 g l**^**-1**^**)**	1.58	1.75	1.90	**γ*l* (1 day)**
	2.03	2.03	2.03	**γ*m* (2 day)**
	2.40	2.43	2.45	**γ*h* (3 day)**
**Full depletion of aromatic metabolites by *P. putida* (days)**				
**α*l* (10 g l**^**-1**^**)**	17.95	18.58	26.35	**γ*l* (1 day)**
	22.18	21.50	29.68	**γ*m* (2 day)**
	26.30	24.20	32.70	**γ*h* (3 day)**
**α*m* (14 g l**^**-1**^**)**	15.35	17.05	24.43	**γ*l* (1 day)**
	19.63	20.15	27.98	**γ*m* (2 day)**
	24.13	23.15	31.40	**γ*h* (3 day)**
**α*h* (20 g l**^**-1**^**)**	12.90	15.45	22.53	**γ*l* (1 day)**
	17.00	18.68	26.25	**γ*m* (2 day)**
	21.68	21.95	29.98	**γ*h* (3 day)**

(**α**) Symbol to identify glucose concentration.

(**β**) Symbol to identify AR27 dye concentration.

(**γ**) Symbol to identify bacterial inoculation time.

(***l)*** Low level.

(***m***) Medium level.

(***h***) High level.

Initial experimental results showed that pure cultures of *T. versicolor* decolorized 0.03 g l^-1^ of AR27 in liquid medium in 192 h [44] and in 340 h for an AR27 concentration of 0.2 g l^-1^ [43], while in consortium with *P. putida* the decoloration time of 0.2 g l^-1^ of AR27 was 210 h [43]. This demonstrated that the microbial consortium shows better results in the decoloration of AR27 compared to the decoloration with pure cultures.

**Fig. 4:**
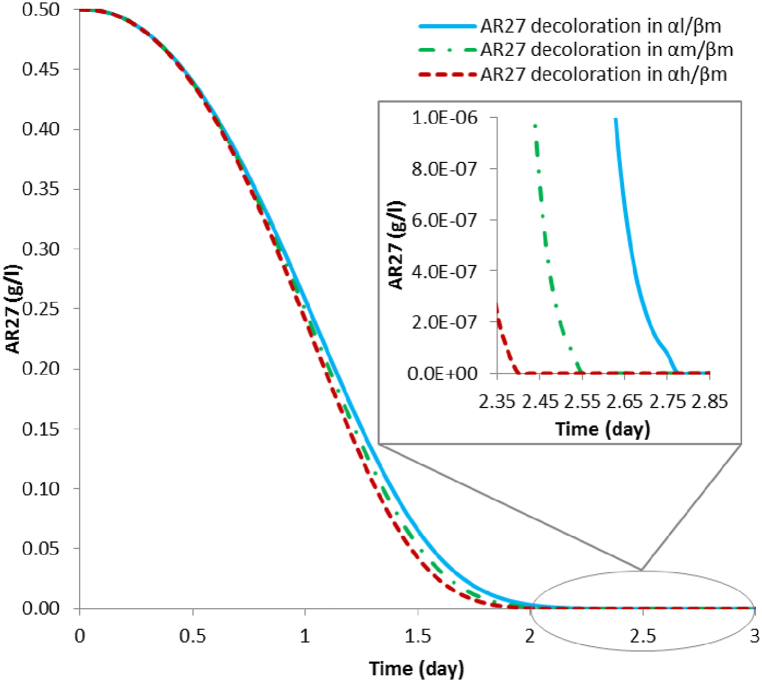
Simulation results of full decoloration of 0.5 g l^-1^ of AR27 by laccase produced with a *T. versicolor* and *P. putida* consortium in scenarios **α*l*/β*m***; **α*m*/β*m***; **α*h*/β*m***.

Fig. 4 shows the decoloration simulations for the scenarios involving **(β*m*)**. In the **α*l*/β*m*** scenarios, complete decoloration of AR27 occurs with an associated laccase activity of 425.53 Ul^−1^; for the **α*m*/β*m*** scenario complete decoloration of AR27 occurs with an associated laccase activity of 491.88 Ul^−1^; and for the **α*h*/β*m*** scenario complete decoloration of AR27 occurs with an associated laccase activity of 534.12 Ul^−1^.

**Fig. 5:**
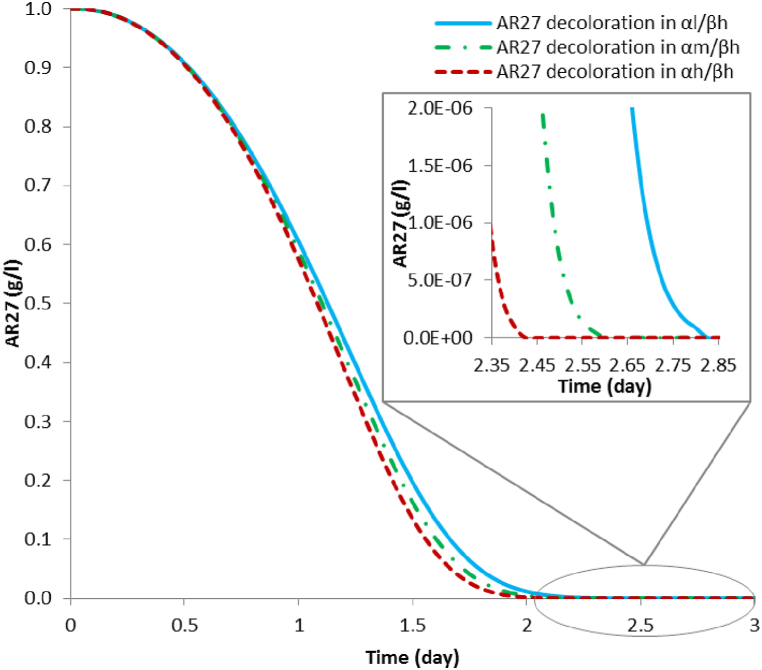
Simulation results of full decoloration of 1.0 g l^-1^ of AR27 by laccase produced with a *T. versicolor* and *P. putida* consortium in scenarios **α*l/*β*h***; **α*m/*β*h***; **α*h/*β*h***.

Fig. 5 shows the decoloration simulations of 1.0 g l^-1^ AR27 for the three (**β*h*)** cases. In the **α*l*/β*h*** scenarios complete decoloration of AR27 occurs with an associated laccase activity of 439.99 Ul^−1^; for the **α*m*/β*h*** scenarios complete decoloration of AR27 occurs with an associated laccase activity of 517.34 Ul^−1^; and for the **α*h*/β*h*** scenarios complete decoloration of AR27 occurs with an associated laccase activity of 550.67 Ul^−1^.

The results obtained prove stability of the mathematical model by consistently simulating, for each scenario, such as: the longer decoloration times and higher laccase activity required according to the increase of the initial AR27 concentration. Similarly, the high affinity of the laccase enzyme for the AR27 dye is theoretically proven by simulating similar decoloration times, in all cases, and short variations with respect to the associated laccase activity. Namely, the variation in the decoloration time of 0.2 g l^-1^ of AR27 is only 0.2 days respect to the scenarios (**α*l*/β*l*** and **α*m*/β*l***) and 0.15 days for the scenarios (**α*m*/β*l*** and **α*h*/β*l***); on the other hand, for the decoloration of 0.5 g l^-1^ of AR27, the time variation is 0.23 days for scenarios (**α*l*/β*m*** and **α*m*/β*m***) and 0.15 days for scenarios (**α*m*/β*m*** and **α*h*/β*m***); finally, for the decoloration of 1.0 g l^-1^ of AR27, the variation for scenarios (**α*l*/β*h*** and **α*m*/β*h***) is 0.23 days and 0.18 days for scenarios (**α*m*/β*h*** and **α*h*/β*h***). Concerning the laccase activity required for decoloration, the average for all simulations is 485.32 Ul^−1^ and the variation between the highest and lowest enzyme requirement scenario does not exceed 140.00 Ul^−1^.

### 3.3 Production/consumption of metabolites and bacterial growth

Simultaneously to the decoloration of AR27 by the enzymatic action of the laccase excreted by *T. versicolor*, which causes the production of intermediate metabolites, degradation occurs by the metabolic action of *P. putida*, which is inoculated after the fungal activity was initiated. This phenomenon suggests an interaction of co-metabolism and symbiosis between both microorganisms during the decoloration of AR27.

Simulation of scenarios consider the *P. putida* inoculation strategy reported by González-Reyes (2018) [43], where it is established, that bacterial inoculation is carried out once decoloration of AR27 by T*. versicolor* laccase has started; with this strategy the competition for glucose substrate is avoided during enough time to maintain fungal growth. Likewise, the inoculation strategy allows *P. putida* growth by using aromatic metabolites as their second carbon source once the glucose has been fully depleted. This phenomenon is well known as Catabolite Repression Control (CRC) and it can be described as a diauxic (sequential) growth, in which bacterial growth starts with the uptake of a simple carbon source (such as glucose) until it is completely depleted. During this first growth phase, the enzymes required for the second carbon source (such as aromatic compounds) are repressed. Hence, the CRC is abrogated as the first and simple carbon source approaches depletion. Then, the catabolic machinery required to assimilate the second and complex carbon source is established and the second growth phase begins [100].

**Fig. 6:**
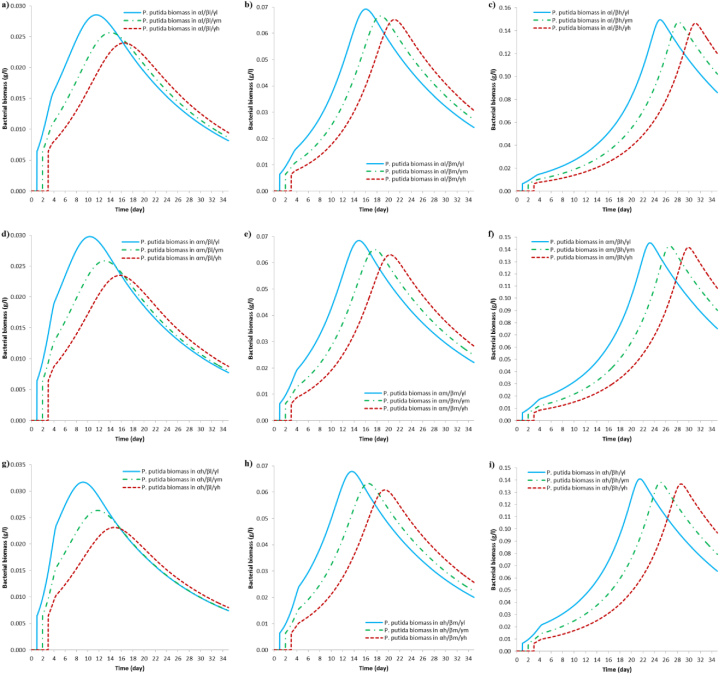
Simulation results of *P. putida* cell growth at different inoculation moments in time, during decoloration of AR27, in scenarios involving (**α*l***), (**α*m***) and (**α*h***). (a), (b) and (c) correspond to scenarios (**α*l/*β*l/*γ*l-m-h***), (**α*l/*β*m/*γ*l-m-h***) and (**α*l/*β*h/*γ*l-m-h***), respectively. (d), (e) and (f) correspond to scenarios (**α*m/*β*l/*γ*l-m-h***), (**α*m/*β*m/*γ*l-m-h***) and (**α*m/*β*h/*γ*l-m-h***), respectively. (g), (h) and (i) correspond to scenarios (**α*h/*β*l/*γ*l-m-h***), (**α*h/*β*m/*γ*l-m-h***) and (**α*h/*β*h/*γ*l-m-h***), respectively.

Fig. 6 shows the results of simulations for cell growth of *P. putida* in consortium with *T. versicolor.* According to this simulation, the bacterial growth is affected by the three independent variables that have been defined, mainly by the moment in time at which *P. putida* is inoculated, followed by the initial concentration of AR27. All the scenarios seen in Fig. 6 (a – i) show that cell growth of *P. putida* is higher when inoculated on day 1 and decreases when inoculated on days 2 and 3. This is due to the fact that when inoculated on day 1, when the availability of glucose is high for both microorganisms, *P. putida* grows consuming the remaining glucose as its first carbon source (first growth phase) and then continue the second growth phase with the consumption of aromatic compounds as a second carbon source [100], which at the same time are generated until they reach their maximum concentration, depending upon the initial concentration of AR27. These results suggest that when inoculating the bacteria on day 1, there may be competition for the glucose which would not be beneficial for fungal growth and enzyme production for AR27 decoloration.

The same pattern is noted when the bacterium is inoculated on day 2 for all scenarios shown in Fig. 6 (a - i). In this case, the availability of glucose is lower, so the bacterium could grow by two ways: (i) by consuming the glucose as a first carbon source (first growth phase) and then continue the second growth phase with the consumption of aromatic compounds as a second carbon source [100] or (ii) by preferably consuming the aromatic metabolites present in the medium [61], which have not yet reached their maximum concentration. When the bacterium is inoculated on day 3 [see Fig. 6 (a – i)], the available glucose is about to be depleted and the aromatic metabolites have already reached their maximum concentration, so *P. putida* grows by consuming aromatics as a primary carbon source, additionally it has also been reported that *P. putida* CSV86 grows preferentially using aromatic compounds instead of glucose due to factors such as: the inability of glucose to suppress enzymes for aromatic degradation and/or the suppression of enzymes for glucose utilization due to the presence of aromatic compounds [61].

Bacterial biomass increases as the initial glucose concentration increases, and especially when the initial AR27 concentration is higher, shown in Fig. 6 (a, d, g) respect to Fig. 6 (b, e, h) and Fig. 6 (c, f, i). The decay in bacterial growth begins when the availability of intermediate metabolites is minimal. Bacterial growth results are summarized in Table 3.

**Fig. 7:**
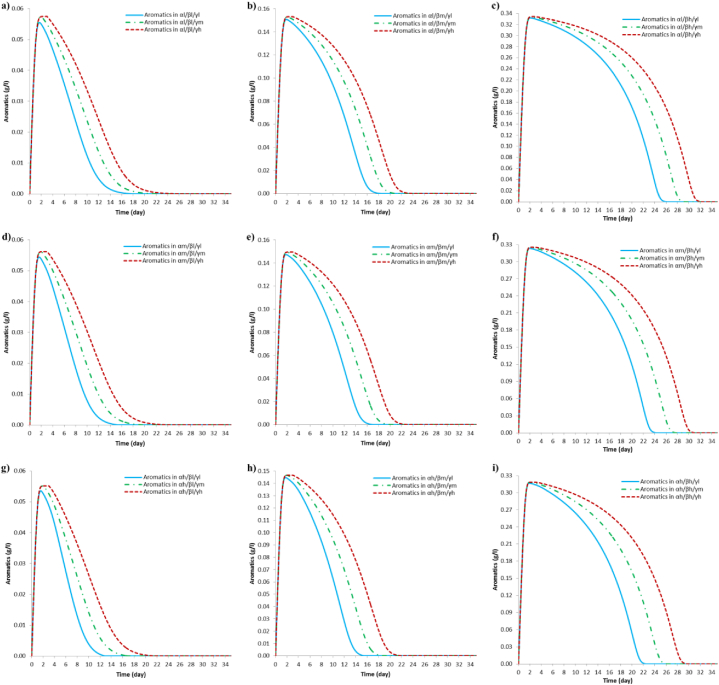
Simulation results of the production and full depletion of aromatic metabolites during decoloration of AR27 in scenarios involving (**α*l***), (**α*m***) and (**α*h***). (a), (b) and (c) correspond to scenarios (**α*l/*β*l/*γ*l-m-h***), (**α*l/*β*m/*γ*l-m-h***) and (**α*l/*β*h/*γ*l-m-h***), respectively. (d), (e) and (f) correspond to scenarios (**α*m/*β*l/*γ*l-m-h***), (**α*m/*β*m/*γ*l-m-h***) and (**α*m/*β*h/*γ*l-m-h***), respectively. (g), (h) and (i) correspond to scenarios (**α*h/*β*l/*γ*l-m-h***), (**α*h/*β*m/*γ*l-m-h***) and (**α*h/*β*h/*γ*l-m-h***), respectively.

Concerning the production of aromatic metabolites, results are summarized in Table 4. These simulations show that the highest concentration of aromatic metabolites is generated with the highest dye concentration; however, aromatic metabolites concentration decreases as the initial glucose concentration increases. This result shows that the scenarios involving (**α*l*/β*h***) have the highest metabolite concentration (Fig. 7c). Regarding simulated scenarios, when *P. putida* is added at day 1 (**γ*l***) and 2 (**γ*m***), it can be noticed that the maximum concentration of intermediate aromatic metabolites is achieved at a further time due to the fact that the AR27 dye has not completely decomposed and bacterial growth is in first phase by consuming the remaining glucose. When the azo dye is fully depleted the concentration of aromatic metabolites remains constant; then decoloration products begin to be taken up by the bacteria until they are fully depleted either by sequential consumption (as a second carbon source) or by preferential consumption upon glucose. A particular case occurs when bacteria are inoculated at day 3 (**γ*a***), in this the maximum concentration of aromatic metabolites is achieved before *P. putida* is inoculated. This phenomenon can be explained by assuming that residual dye is in trace concentrations and, therefore, the concentration of aromatic metabolites remains constant until day 3, when *P. putida* is added and begins to consume aromatics as its main carbon source.

Regarding the consumption of aromatic metabolites (see Table 4) it is interesting that for the three evaluated AR27 concentrations, aromatic metabolites consumed by *P. putida*, inoculated at day 3 (**γ*h***), would take the longest time to decay; whereas the same aromatic metabolites are fully degraded in less time when the bacteria are inoculated on the first day (**γ*l***). This behavior can be seen in the scenarios shown in Fig. 7 (a - i) and it shows the effect of the initial glucose concentration, which allows bacteria to grow sufficiently to later degrade the metabolites formed after decoloration and, in this case, there is an effect from the time of inoculation on the whole degradation rate.

It is important to mention that in the initial experimental assays, the growth of *P. putida* has been carried out using glucose as a carbon source, so a comparative discussion with the results of the mathematical model is not required.

Future work must consider the quantitative characterization of decoloration intermediates and their further degradation by *P. putida*, for this, gas chromatography is an important analytical technique that has provided support for the monitoring of metabolites during biodegradation of textile wastewater [101,102].

Due to the complex composition of textile wastewater, the use of microbial consortia is presented as a viable alternative due to the metabolic mechanism of the organisms involved that can attack the complex chemical structure of azo dyes in different ways to accelerate degradation [103]. Microbial consortia can be built with bacteria, fungi, plants or a mixture of organisms [29]. Some researches have reported complete decolorization of azo dyes by microbial consortia, such as Balapure et al. (2014) [88] who demonstrated, by isolating and immobilizing a bacterial consortium composed of *Alcaligenes* sp. BDN1, *Bacillus* sp. BDN2, *Escherichia* sp. BDN3, *Pseudomonas* sp. BDN4, *Provedencia* sp. BDN5, *Acinetobacter* sp. BDN6, *Bacillus* sp. BDN7 and *Bacillus* sp. BDN8, 100 % decoloration from 100 ppm azo blue reactive dye 160 in 4 h. Meanwhile, the study by Wang et al. (2018) [104] showed that the bacterial consortium composed of *Paludibacter*, *Trichococcus* and *Methanosarcina* decolored 97.2 % from 100 ppm of the reactive red azo dye 2 in 4 h. Another bacterial consortium composed of *Pseudomonas, Lysinibacillus, Lactococcus* and *Dysgonomona* achieved more than 85 % decoloration from 300 ppm of the azo dye methanil yellow in 6 h, according to the study by Guo et al. (2019) [105]. Another bacterial consortium, composed of *Citrobacter freundii* A1, *Enterococcus casseliflavus* C1 and *Enterobacter cloacae* L17, fully decolored 100 ppm of acid red 27 in 30 min, according to the study by Chan et al. (2012) [106]. Microbial consortia of different species have also been reported. In (2004), He et al. [107] studied a consortium composed of a white rot fungus and a bacterium, *Pseudomonas* sp. to demonstrate 99.1 % of decoloration from 150 ppm of the direct azo red dye 23 in 24 h. The microbial consortium composed of the fungus *Penicillium* sp.QQ and the bacterium *Sphingomonas xenophaga* QYY was studied to demonstrate 87.8 % decoloration from 50 ppm of two azo dyes, reactive red 2 and acid red B 14, in 72 h by Gou et al. (2009) [108]. Another consortium composed of a fungus and a bacterium, *Providencia rettgeri* HSL1 and *Aspergillus ochraceus* NCIM 1146 was studied in (2016) by Lade et al. [109] who demonstrated degradation of 100 ml from real wastewater by 92 % in 30 h. In (2013), Kabra et al. [110] studied a microbial consortium composed of the bacterium *Pseudomonas monteilii* ANK and the plant *Glandularia pulchella* (Sweet) Tronc. to demonstrate 92 % decoloration/biotransformation from 50 ppm synthetic textile waste effluent composed of remazol red, malachite green, scarlet RR, direct red 2B and brown 3 REL. Another study by Kabra et al. (2011) [111] demonstrated the decoloration capacity of the plant consortium composed of *Aster amellus* Linn. and *Glandularia pulchella* (Sweet) Tronc. from 20 ppm of the azo dye orange remazol 3R in 36 h.

Based on the literature review conducted for the present work, no study of decoloration and/or biodegradation of the acid red azo dye 27 by the microbial consortium formed by *T. versicolor* and *P. putida* has been reported, so this study provides an approximation of the capacity of the novel consortium for the decoloration and degradation of azo dyes. In turn, the increase of publications reporting textile dye bioremediation research since 2000 is an indicative that biological methods of textile wastewater treatment have operational advantages over physicochemical treatment methods with respect to cost and decoloration/degradation efficiency [112].

Preliminary, it is believed that the microbial consortium presented and described in this work has potential applicability to other types of dyes given the metabolic capacity of the species involved. For example, it has been shown that *Trametes versicolor* BAFC 2234 showed decoloration rates of 97 % for the triphenylmethane dye malachite green and 52 % for the azo dye xylidine in 240 min in a two-phase bioreaction system [113]. Another study showed the ability of *Trametes versicolor* CBR43 to decolorize azo and anthraquinone-type dyes, as it decolored more than 90 % from 200 ppm of acid dyes such as red 114, blue 62 and black 172, as well as reactive dyes such as red 120, blue 4, orange 16 and black 5 in 6 days. This research also demonstrated 67 % decoloration from 200 ppm acidic orange 7 in 9 days, while the decoloration efficacies of disperse dyes such as red 1, orange 3, and black 1 were 51–80 % in 9 days [114]. The study by Casas et al. (2009) [69] showed that *Trametes versicolor* can decolorize triphenylmethane dyes such as brilliant green 1 (90, 79 and 76 % for concentrations from 25, 75 and 105 ppm, respectively) and basic fuscin (97, 95 and 77 % for concentrations from 50, 100 and 140 ppm, respectively) after 48 h. In turn, *P. putida* bacteria are capable of degrading aromatic compounds through established metabolic pathways, which gives them a wide utility in biodegradation processes of pollutants [97], such as phenol [[115], [116], [117], [118]], 2,4-dichlorophenol [119], mixtures of phenol and catechol [53], mixtures of benzene, toluene and phenol [120,121], etc. This suggests that the microbial consortium proposed in this work may have the ability to decolorize and biodegrade other types of textile dyes under the conditions studied.

Initial experimental results were evaluated and compared with model results, finding general similarities in the behavior of the system and the model predictions, the most representative of which are summarized in Table 5.

**Table 5:** Comparison of initial experimental results and mathematical model prediction.

Characteristic	Initial experimental result	Mathematical model prediction
Maximum microbial biomass	Maximum fungal biomass generation was reached at 158 h and maximum bacterial biomass at 12 h in pure cultures. After 10 h of bioreaction, no bacteria were detected in the consortium culture medium. An analysis at 130 h of bioreaction allowed determining the presence of bacterial cells in the fungal hyphae, which improved the production of fungal pellets [43].	Maximum fungal biomass at 97.2 h and maximum bacterial biomass at 377.52 h.
Glucose consumption	At 123 h, 75 % of the initial glucose (4 g l^-1^) has been consumed and it is fully depleted at 240 h [43].	Simulation shows consumption above 95 % at 97.2 h. Full consumption at 98.4 h.
Laccase activity	Maximum laccase activity (851.8 Ul^−1^) was detected at 168 h [43].	Maximum laccase activity (1168.81 Ul^−1^) was simulated at 93.12 h.
AR27 decoloration	Complete AR27 decoloration at 192 h (0.03 g l^-1^) [44] and 210 h (0.2 g l^-1^) [43].	The simulation predicted full decoloration at 60.72 h (0.2 g l^-1^).

### 3.4 Sensitivity analysis by Response Surface Method

It was analyzed the effect of three factors (independent variables): initial glucose concentration (**α**), initial AR27 concentration (**β**) and inoculation time of *P. putida* (**γ**); on three responses of interest (dependent variables): decoloration of AR27 (**R**_**1**_), maximal production of aromatic metabolites (**R**_**2**_) and degradation of aromatic metabolites (**R**_**3**_). Each factor was evaluated at three levels: low, medium, and high. The responses were analyzed in terms of the minimum time (in days) to reach full production/consumption. Thus, conditions for **R**_**1**_ as the minimum decoloration time were estimated; for **R**_**2**_ the minimum time in which the maximum production of aromatic metabolites is achieved, and for **R**_**3**_ the minimum time in which aromatic metabolites are degraded. The sensitivity analysis was carried out with the aim of proposing the theoretic local optimum conditions for the bio reaction process. The coefficients of the corresponding polynomials were obtained by performing a sensitivity analysis in Microsoft Excel® 2010 using multivariate regressions.

**Fig. 8:**
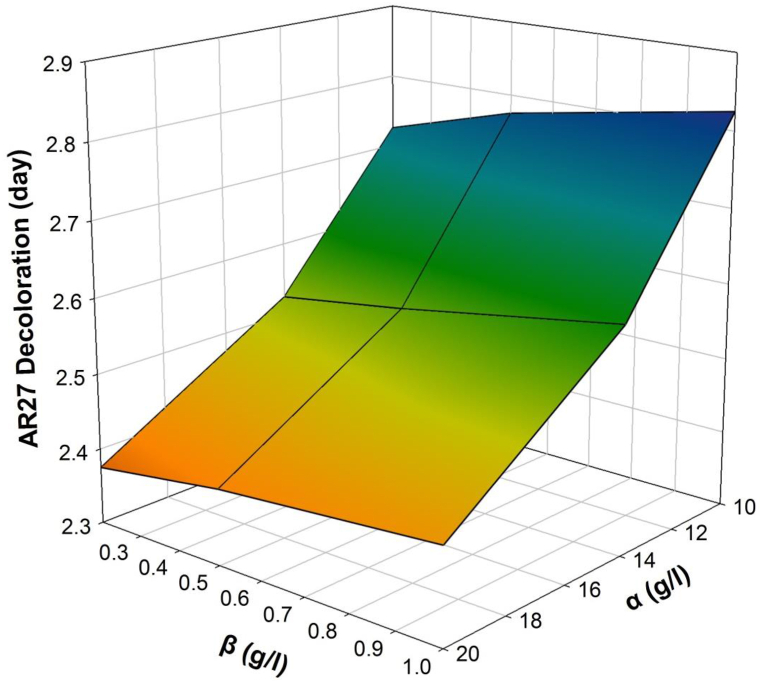
Response surface for identifying the minimum time of full AR27 decoloration time (**R**_**1**_). The interactive effect between the initial AR27 concentration (**β**) and the initial glucose concentration (**α**) is shown.

The sensitivity analysis for the minimum time of full AR27 decoloration (**R**_**1**_) enabled us to determine that the factor (**γ)** (inoculation time of *P. putida*) has the lowest effect on the response of interest, so the response surface was calculated only using the variables (**α)** and (**β)**. In this sense, Fig. 8 shows that the effect of the initial concentration of glucose (**α**) upon the decoloration time of AR27 is inversely proportional since it was predicted that the higher the initial concentration of glucose, then, the shorter is the decoloration time of AR27. On the other hand, the initial AR27 concentration (**β**) is directly proportional to the time taken for its decoloration, i.e., the higher the initial AR27 concentration, the longer it will take for full decoloration. In that sense, the minimum time of full AR27 decoloration is the one corresponding to the (**α*h*/β*l***) scenarios. The quadratic polynomial for **R**_**1**_ is shown in equation (10), which has a correlation coefficient of r^2^ = 0.9996.

(10) $$R_{1}=3.5933-0.1174\alpha +0.2245\beta +0.0028\alpha^{2}-0.0347\beta^{2}-0.0061\alpha \beta$$

**Fig. 9:**
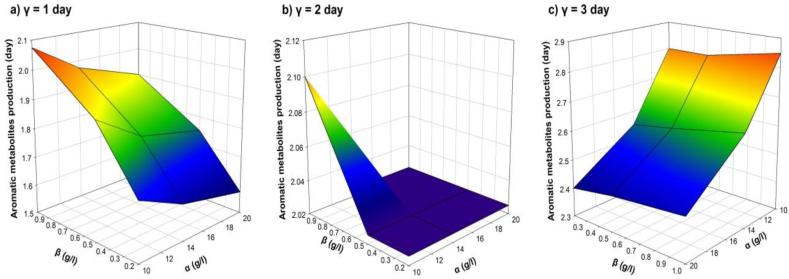
Response surfaces for the minimum time of maximum aromatic metabolite production (**R**_**2**_). Interactive effects between the initial glucose concentration (**α**), initial AR27 concentration (**β**) and *P. putida* inoculation moment in time (**γ**) are shown for (a) 1 day, (b) 2 days and (c) 3 days.

Sensitivity analysis respect to the minimum time for maximum aromatic metabolites production (**R**_**2**_) showed that the strongest factor with the major influence upon this response is the initial AR27 concentration (**β**), followed by the interaction between the initial AR27 concentration and the time for bacterial inoculation (**βγ**); The latter with an inverse effect upon **R**_**2**_ as it happened with the initial glucose concentration (**α**). Fig. 9 shows the response surface plots for **R**_**2**_ with the interactions between the three factors. Fig. 9a shows the response surface when *P. putida* is inoculated at day 1; Fig. 9b is the response surface when *P. putida* is inoculated at day 2; and Fig. 9c is the response surface when *P. putida* is inoculated at day 3. For each case as the initial concentration of AR27 increases, the time in which the maximum concentration of aromatic metabolites is achieved is longer. This phenomenon is repeated with respect to the time in which *P. putida* is inoculated. On the other hand, at higher initial glucose concentrations, the maximum concentration of aromatic metabolites is achieved in a shorter time. The minimum time for maximum aromatic metabolites production is the one corresponding to the (**α*h*/β*l*/γ*l***) scenario. The quadratic model for **R**_**2**_ shown in equation (11) and it was the best adjusted to the data with a correlation coefficient of r^2^ = 0.973.

(11) $$\begin{array}{c} R_{2}=1.8199-0.0425\alpha +0.7983\beta -0.0015\gamma +0.0018\alpha^{2}-0.0764\beta^{2}+\cdots \\ \cdots +0.1736\gamma^{2}-0.0116\alpha \beta -0.0133\alpha \gamma -0.2144\beta \gamma +0.0026\alpha \beta \gamma \end{array}$$

**Fig. 10:**
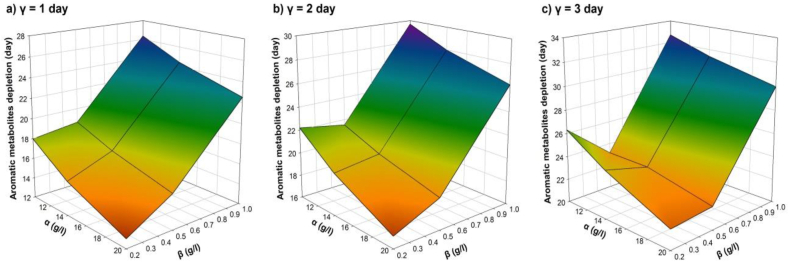
Response surfaces for the minimum time of full depletion of aromatic metabolites (**R**_**3**_). Interactive effects, between: the initial glucose concentration (**α**), the initial AR27 concentration (**β**) and the moment in time of inoculation of *P. putida* (**γ**) are shown for (a) 1 day, (b) 2 days and (c) 3 days.

According to the sensitivity analysis for the minimum time of full depletion of aromatic metabolites (**R**_**3**_), the model that showed the highest correlation coefficient was the quadratic model (r^2^ = 0.9909), which is shown in equation (12). For this case, the strongest factor with the major influence upon this response is the quadratic coefficient of the initial AR27 concentration **(β**^**2**^**)** that indicates the dependence of the degradation time of aromatic metabolites on initial dye concentration, which describes that the higher the dye concentration, the longer the degradation time. The moment in time in which the bacteria are inoculated (**γ**) also has a direct influence on the degradation of metabolites: the longer the inoculation time, the longer it will take to eliminate the intermediate compounds. In turn, the initial glucose concentration (**α**) has an inverse influence. In this case, the higher the initial glucose concentration, then it is the shorter time for a full degradation of aromatic metabolites. The minimum time for a full depletion of aromatic metabolites is the one corresponding to the (**α*h*/β*l*/γ*l***) scenario. Fig. 10 shows the response surface plots for **R**_**3**_ where Fig. 10a shows the response surface when *P. putida* is inoculated at day 1; Fig. 10b is the response surface when *P. putida* is inoculated at day 2; and Fig. 10c is the response surface when *P. putida* is inoculated at day 3.

(12) $$\begin{array}{c} R_{3}=23.1037-0.9056\alpha -10.7569\beta +3.8688\gamma +0.0137\alpha^{2}+17.9375\beta^{2}+\cdots \\ \cdots -1.6817{\times 10}^{-16}\gamma^{2}+0.0871\alpha \beta +0.0157\alpha \gamma -1.4677\beta \gamma +0.0406\alpha \beta \gamma \end{array}$$

The analysis of these results allows us to propose an “ideal scenario” for the decoloration and degradation of AR27 by the microbial consortium of *T. versicolor* and *P. putida*. According to the study of each case, the (**α*h*/β*l*/γ*l***) scenario, corresponding to the following conditions: 20 g l^-1^ of initial glucose, 0.2 g l^-1^ of initial AR27 and a bacterial inoculation at day 1, is to get the best decoloration time of AR27, a maximum production of aromatic metabolites and their subsequent degradation in the minimum possible time, therefore this is proposed as the local optimum one. However, the (**α*h*/β*l*/γ*m***) scenario could be considered to avoid a competence for the substrate, as described by Gonzalez-Reyes (2018) and Cortazar-Martínez (2013) [43,44]. In that sense, the effect of the initial concentration of microbial biomass would be subject to future analyses.

## 4 Conclusions and future perspectives

An analysis of the process that follows the decoloration and degradation of the azo dye acid red 27 (AR27) by a novel microbial consortium of *T. versicolor* and *P. putida* was performed through simulation of scenarios with different operational conditions by using the proposed mathematical model that was developed and based on the kinetic equations of Monod, Haldane-Andrews and Michaelis-Menten. The mathematical model was able to simulate the behavior of the two microbial species during the bio-reaction process at different operative conditions; focusing, in a first stage, on the decoloration of AR27 by the enzymatic reaction of laccase produced by *T. versicolor*, followed by a second stage of degradation of aromatic metabolites by metabolic action of *P. putida*. The sensitivity analysis performed by applying the Response Surface Method made it possible to determine the individual and interactive effect of independent variables over the response variables of interest, which allows proposing the theoretically local optimum conditions for the reaction process, being: 20 g l^-1^ of initial glucose concentration, 0.2 g l^-1^ of initial AR27 concentration and an inoculation moment in time of *P. putida* at day 1. Results of this study suggest that as a first approximation, the use of mathematical modeling is a feasible tool for designing a bio-reaction system, which can operate with the microbial consortium of *T. versicolor* and *P. putida*, for the decoloration and further degradation of the acid red azo 27 dye. The theoretical local optimum conditions proposed in this study will be used during the operation of the bio-reaction system to be designed. Further optimization of the mathematical model may be necessary. It is expected that the bioreaction system to be designed can be used to demonstrate the applicability of the consortium during the decoloration and further degradation of acid red 27 azo dye as a first stage and serve as a basis for future researches of bioremediation of textile wastewater at pilot scale for future industrial scale-up and to propose the bioremediation system under the principle of multispecies microbial consortia as an alternative technique for a tertiary textile wastewater treatment.

The design and development of the bioreactor system operating with the consortium of *T. versicolor* and *P. putida* may have challenges to be considered and addressed in future research; these should include consideration of factors such as cell growth and maintenance of the species, e.g., providing a continuous carbon source for the growth and maintenance of the consortium; the inoculation technique of both microorganisms, so that the operation of the bioreactor and the survival of the species is not affected. Experimental factors such as operational stability, oxygen transfer, pH and temperature control, scale-up and energy requirements should be considered.

## Data availability statement

No data associated with this study has been deposited into a publicly available repository, then data will be made available on request.

## Additional information

No additional information is available for this paper.

## CRediT authorship contribution statement

**L.A. Martínez-Castillo:** Data curation, Formal analysis, Investigation, Methodology, Software, Writing – original draft, Writing – review & editing. **C.A. González-Ramírez:** Conceptualization, Data curation, Formal analysis, Funding acquisition, Investigation, Methodology, Project administration, Resources, Supervision, Writing – original draft, Writing – review & editing. **A. Cortazar-Martínez:** Investigation, Methodology, Software. **J.R. González-Reyes:** Data curation, Formal analysis, Investigation, Methodology, Visualization. **E.M. Otazo-Sánchez:** Formal analysis, Supervision, Visualization. **J.R. Villagómez-Ibarra:** Formal analysis, Investigation, Supervision, Validation, Visualization, Writing – review & editing. **R. Velázquez-Jiménez:** Formal analysis, Supervision, Visualization. **G.M. Vázquez-Cuevas:** Formal analysis, Resources, Supervision, Visualization. **A. Madariaga-Navarrete:** Resources, Supervision, Visualization. **O.A. Acevedo-Sandoval:** Resources, Visualization. **C. Romo-Gómez:** Resources, Visualization.

## Declaration of competing interest

The authors declare that they have no known competing financial interests or personal relationships that could have appeared to influence the work reported in this paper.
